# Resveratrol Improves Memory and Reduces Neuroinflammation in an MPS IIIB Mouse Model

**DOI:** 10.1007/s11064-026-04920-6

**Published:** 2026-10-13

**Authors:** Maja Ziemian, Grażyna Jerzemowska, Joanna Szmydtka, Lidia Gaffke, Szymon Wojciechowski, Zuzanna Cyske, Grzegorz Węgrzyn, Estera Rintz

**Affiliations:** 1 Department of Molecular Biology, Faculty of Biology, University of Gdańsk, Gdańsk, Poland; 2 Department of Animal and Human Physiology, Faculty of Biology, University of Gdańsk, Gdańsk, Poland

**Keywords:** Mucopolysaccharidosis, Immune response, Behavioral test, Memory deficit, Sex difference

## Abstract

Mucopolysaccharidosis type IIIB (Sanfilippo syndrome type B) is a neurodegenerative lysosomal storage disorder characterized by cognitive decline, behavioral abnormalities, and neuroinflammation, for which no effective central nervous system (CNS)-targeted therapy currently exists. Increasing evidence indicates that inflammatory processes contribute substantially to disease progression. In this study, we evaluated the effects of resveratrol on behavioral outcomes and neuroinflammatory markers in an MPS IIIB mouse model, including both sexes. Behavioral analyses demonstrated anxiety-like behavior and memory impairment in MPS IIIB mice. Resveratrol treatment improved cognitive performance, reduced anxiety-related behavior, and normalized exploratory activity. Premature deaths occurred only in untreated MPS IIIB mice. Hematological and biochemical analyses revealed systemic inflammation, erythropoietic stress, metabolic dysregulation, and altered liver-associated parameters. Diseased mice exhibited leukocytosis, elevated pro-inflammatory immune cell populations, increased globulin concentrations, disturbed glucose and lipid metabolism, and sex-dependent alterations in liver and renal markers. Resveratrol partially normalized these abnormalities and reduced inflammatory and hematological disorders. Peripheral and central inflammatory responses were assessed by measurement of IL-1β, IL-6, and c-Fos levels. MPS IIIB mice displayed widespread neuroinflammation in the hippocampus, cortex, and amygdala, with pronounced sex- and region-dependent differences. Females exhibited stronger inflammatory changes in CA1, CA2, and retrosplenial cortical regions, whereas males showed predominant involvement of CA3, dentate gyrus, and motor cortical areas. Resveratrol reduced pro-inflammatory cytokine production and normalized neuronal hyperactivity, particularly in males. Overall, resveratrol modulated neuroinflammatory markers and improved selected behavioral outcomes in MPS IIIB mice, suggesting its potential to ameliorate CNS-related manifestations of Sanfilippo syndrome.

**Supplementary Information:**

The online version contains supplementary material available at https://doi.org/10.1007/s11064-026-04920-6.

## Introduction

Mucopolysaccharidoses (MPS) are a group of rare lysosomal storage disorders caused by impaired degradation of glycosaminoglycans. One of the most common types is MPS III (Sanfilippo syndrome), with an estimated incidence of about 0.21 per 100,000 live births. It has five subtypes (A–E), though subtype E has only been observed in animal models. In humans, subtype B is the second most common MPS III and involves disrupted heparan sulfate (HS) degradation due to low or no activity of alpha-N-acetylglucosaminidase enzyme (NAGLU) [1]. HS accumulates in lysosomes, negatively affecting the homeostasis of these organelles. Their membranes become more permeable, leading to a cascade of events such as changes in lysosomal pH, which disrupt the activity of other enzymes and result in the secondary accumulation of additional molecules. Increased membrane permeability further exacerbates these processes, amplifying lysosomal dysfunction.

The permeability of the lysosomal membrane leads to the release of partially degraded HS and other molecules (calcium and iron ions, cathepsins) into the cytosol. This triggers the activation of multiple stress-related pathways. Cathepsin B in the cytoplasm can induce apoptosis and promote inflammation via the inflammasome and cytokines such as IL-1β and IL-18. Released calcium may overload mitochondria, leading to oxidative stress [2]. In turn, released glycosaminoglycans can bind to Toll-like receptor 4 (TLR4), activating the innate immune response. Consequently, immune cells begin to secrete pro-inflammatory cytokines and chemokines, which can spread throughout the body via the vagus nerve or cross the blood–brain barrier, especially as inflammation compromises its integrity [3]. Cytokines act, among others, by binding to receptors on other immune cells and activating them, thereby sustaining systemic inflammation.

In the brain, this primarily leads to microglial activation, as microglia are closely linked to the immune system. As a result, inflammation in the central nervous system (CNS) is intensified, exerting a degenerative effect on neurons. This includes impaired synaptogenesis, disrupted axonal transport and myelination, and dendritic abnormalities [2, 4–6].

HS plays a particularly important role in the brain, as it is essential for the proper formation and maintenance of neuronal network architecture and for neurotransmission. This is due to its involvement in numerous signaling pathways, from neurogenesis, through axonal guidance, to synaptogenesis [7]. Consequently, HS indirectly regulates processes related to short- and long-term memory, motor function, social behavior, sleep, and emotions. Therefore, in MPS IIIB patients, symptoms affecting the central nervous system are the most severe. The first symptoms appear between 1 and 4 years of age and include developmental delay. Over time, cognitive impairment worsens, accompanied by behavioral and motor disturbances that become increasingly prominent. Around adolescence, patients undergo regression, with development plateauing at the level of approximately a 3-year-old child [8]. Somatic symptoms such as hepatosplenomegaly, skeletal deformities, and hearing impairment also occur but are generally milder than in other types of MPS [9]. Depending on disease progression, patients typically die between the second and third decade of life [10].

Currently, no effective treatment is available for patients with MPS III. An ideal therapeutic approach should be able to cross the blood–brain barrier and modulate the strong immune response both in the CNS and in peripheral tissues. Our previous studies have shown that resveratrol, a natural polyphenolic compound, fulfills these criteria [11]. It exhibits multiple beneficial properties, including anti-inflammatory, anti-apoptotic, and antioxidant effects, making it an increasingly promising candidate for therapeutic applications, particularly in neurodegenerative diseases. Its effects are primarily mediated through modulation of molecular pathways. We previously demonstrated that resveratrol stimulates autophagy via SIRT1, mTOR, and FOXO3 signaling pathways, which are particularly important since lysosomal dysfunction caused by HS accumulation is a key driver of MPS IIIB pathology [12].

Resveratrol also shows strong potential in combination therapies, including with enzyme replacement therapy [13]. This is largely due to its combined properties, which help alleviate symptoms such as inflammation, promote tissue recovery, and enhance the degradation of accumulated glycosaminoglycans through autophagy. Additionally, it increases the levels of the mannose-6-phosphate receptor, facilitating more efficient delivery of therapeutic enzymes to lysosomes.

In this study, we investigate the effects of resveratrol on memory function in both male and female MPS IIIB mice in relation to immune responses, both in the peripheral system and in the CNS. Specifically, we aimed to determine whether resveratrol-mediated modulation of inflammatory pathways correlates with improvements in cognitive performance and behavioral outcomes, and whether these effects exhibit sex-dependent differences.

## Methodology

### Experimental Model

Male and female homozygous *Naglu* knockout mice (MPS IIIB model), carrying a targeted disruption of the gene encoding α-N-acetylglucosaminidase (B6.129S6-Naglutm1Efn/J; Cat. No. 003827; The Jackson Laboratory, USA), were used in this study. Age-matched healthy littermates (WT; C57BL/6) served as control animals. Mice were housed in a ventilated animal facility under controlled environmental conditions, including a 12 h light/12 h dark cycle, ambient temperature maintained at 22 °C, and relative humidity of approximately 55%. Food and water were available ad libitum throughout the experiment. All procedures involving animals were approved by the Local Ethics Committee for Animal Experiments in Bydgoszcz, Poland (approval no. 13/2020, issued June 25, 2020).

**Fig. 1:**
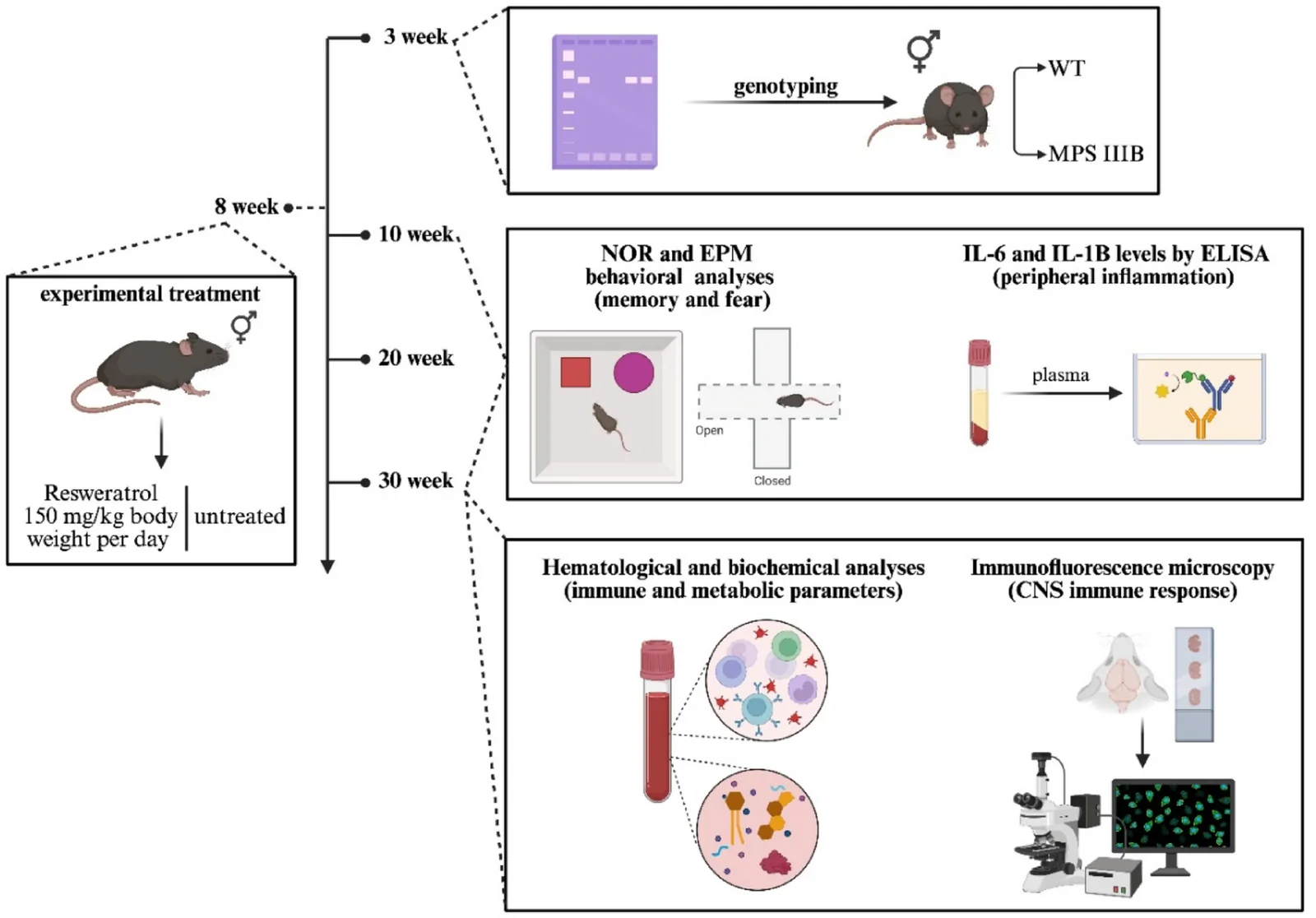
The experimental scheme. Resveratrol or water was administered daily by oral gavage beginning at 8 weeks of age. Behavioral and biochemical assessments were conducted at three experimental time points throughout the study. Animals were euthanized after the final assessment at 30 weeks of age. Created in BioRender. https://BioRender.com/vanuajm

Animals were divided into eight experimental groups based on sex and treatment (four male and four female groups). The groups included: (i) wild-type (WT) mice receiving water (WT), (ii) WT mice treated with resveratrol (WT + R), (iii) MPS IIIB mice receiving water (MPS IIIB), and (iv) MPS IIIB mice treated with resveratrol (MPS IIIB + R). The experimental timeline is shown in Fig. 1. Resveratrol (Sigma-Aldrich, St. Louis, MO, USA) was prepared as a suspension in water and administered orally by metal gavage at a dose of 150 mg/kg body weight per day starting from 8 weeks of age.

### Elevated Plus Maze Test

The elevated plus maze was positioned 50 cm above the floor. Behavioral testing was conducted at a fixed time of day between 2 and 3 PM. Each session was recorded for 5 min using EthoVision XT 10 software (Noldus, Wageningen, Netherlands). At the beginning of the test, mice were placed on one of the open arms facing away from the center of the maze. After completion of the trial, animals were returned to their home cages. To minimize olfactory cues, the maze was cleaned with 70% ethanol between trials and allowed to dry for 5 min before the next recording. The EPM procedure was performed three times during the experimental timeline: under baseline conditions, after completion of the experimental cycle, and as a re-test conducted 60 min later to assess memory-related responses.

### Novel Object Recognition Test

The Novel Object Recognition (NOR) test was used to assess recognition memory and anxiety-related behavior. The test was performed at three time points on a white wooden arena (100 × 100 × 60 cm), with the floor divided into 25 equal squares defining central and peripheral zones. At 6 weeks of age, mice underwent three sessions within a single day (each 10 min), separated by 1-h intervals. The sessions were recorded using EthoVision XT 10 software (Noldus, Wageningen, Netherlands). The arena and objects were cleaned with 70% ethanol between trials to eliminate olfactory cues. The first session was conducted in an empty arena to allow habituation to the environment. During the second session, two identical objects were placed in the center of the arena, and in the third session one familiar object was replaced with a novel one. Mice tested at 20 and 30 weeks of age underwent only two sessions (familiarization and test), without the habituation phase. The analysis included the total exploration time (combined time spent at both objects) and the discrimination index, calculated as (T_novel - T_familiar)/(T_novel + T_familiar) x 100%, where T_novel represents the exploration time of the novel object and T_familiar represents the exploration time of the familiar object.

### Hematological and Biochemical Analysis

Blood samples were collected at the final stage of the experiment (week 30) from the jugular vein. Whole blood was used to determine both hematological with URIT-5160VET (Urit Medical Electronic Co., Shenzhen, China) and biochemical parameters with Exigo C200 (Boule, Spånga, Sweden). Blood samples were then centrifuged to obtain plasma for further analysis and stored at −20 °C.

### Immunological Parameters

Peripheral inflammation was assessed by measuring pro-inflammatory cytokines (IL-1β and IL-6) in plasma using the ELISA method, following the protocol provided by Abcam, from which the kits were obtained (ab222503-1 × 96 for IL-6 and ab197742-1 × 96 for IL-1β). Briefly, plasma samples from mice aged 30 weeks were diluted in a dedicated buffer (Sample Diluent NS) at ratios of 1:4 for IL-1β and 1:6 for IL-6. The samples were incubated with antibodies for 1 h at room temperature on a shaker (400 rpm). Subsequently, the contents of the wells were washed three times with wash buffer, and a substrate solution was added. The samples were then incubated in the dark for approximately 7 min, until the solution changed color from colorless to blue. The reaction was stopped by adding a stop solution, after which absorbance was measured at a wavelength of 450 nm with Varioskan ALF Multimode microplate reader (ThermoFisher). Cytokine concentrations were determined based on a standard curve.

### Brain Immunofluorescence

Immunofluorescence studies were performed as previously described [12] on 20 μm coronal sections obtained from the right hemispheres of mouse brains using a Leica CM1950 cryostat (Leica, Germany). Sections were mounted on gelatin-coated slides (Superfrost™ Plus, Epredia, Netherlands). Staining was conducted to evaluate c-Fos, IL-1β, IL-6, and DAPI labeling. All procedures were performed at room temperature using phosphate-buffered saline (PBS, pH 7.4) containing 0.3% Triton X-100, normal goat serum (NGS), and bovine serum albumin (BSA). Sections were incubated for 48 h with primary antibodies against c-Fos (mouse monoclonal, 1:200; Santa Cruz Biotechnology, Dallas, TX, USA), IL-6 (rabbit polyclonal, #ab6672, Abcam, Cambridge, UK), or IL-1β (rabbit polyclonal, #ab2105, Abcam, Cambridge, UK), followed by 4 h incubation with fluorophore-conjugated secondary antibodies (Invitrogen, Thermo Fisher Scientific). Slides were mounted using DAPI-containing medium (EverBrite Hardset, Biotium). Images were acquired using an Olympus microscope at 20× magnification and processed with CellSens software. Whole sections were additionally imaged using a stereomicroscope (Stemi 508; Zeiss) equipped with an Axiocam 105 color camera and integrated Zen Digital Imaging software (version 3.2 Blue Edition; Zeiss) at 1.65 × 10 magnification with a scale bar of 500 μm. Images of the whole hippocampus were acquired at 3.2 × 10 magnification with a scale bar of 500 μm, whereas higher-magnification micrographs were captured at 20× magnification with a scale bar of 100 μm. Automated threshold-based analysis was used to quantify c-Fos, IL-1β, IL-6, and DAPI-positive cells, expressed as cell density (cells/mm^2^). Detailed analyses were performed in limbic areas, including the cingulate cortex (CG1, CG2), retrosplenial cortex (RSA, RSG), amygdala (Ce, BLA, and BST), and hippocampus (CA1, CA2, CA3, DG), as well as motor cortex areas (M1, M2).

### Statistical Analysis

Data distribution was first examined using the Kolmogorov–Smirnov test, and equality of variances was evaluated with Levene’s test. For datasets meeting assumptions of normality and homoscedasticity, statistical comparisons were performed using one-way or two-way ANOVA followed by Tukey’s post hoc test. When these assumptions were not satisfied, the non-parametric Kruskal–Wallis test was applied, with Dunn’s test used for multiple comparisons. Survival curves were generated using the Kaplan-Meier method and compared using the log-rank (Mantel-Cox) test. All analyses were carried out using GraphPad Prism 9 software, and statistical significance defined as *p* < 0.05. Data are presented as mean ± standard deviation (SD), as indicated in the figure legends. Levels of statistical significance are denoted by asterisks: *p < 0.05, **p < 0.01, ***p < 0. 001, and ****p < 0.0001. Differences between male and female mice are indicated by hashtag: ^#^*p* < 0.05; ^###^*p* < 0.001; ^####^*p* < 0.0001. For behavioral analyses, animals were analyzed at the group level using one-way or two-way ANOVA, depending on the design of the behavioral test. Although the same animals were assessed at different ages, individual mouse IDs were not tracked across time points in the statistical dataset; therefore, repeated-measures analysis was not applied.

## Results 

### Effects of Resveratrol on Mortality and Body Weight in MPS IIIB

MPS IIIB mice have a shortened lifespan in comparison to healthy mice. In the diseased group, two deaths among females and one among males were recorded, whereas no mortality was observed in the treatment group (Fig. S1A). Moreover, male MPS IIIB mice exhibited an increase in body weight, which was normalized to control levels following resveratrol administration (Fig. S1B). These findings indicate lower mortality in the resveratrol-treated group during the experimental period; however, survival analysis revealed no significant differences in survival between the experimental groups. Therefore, although mortality was observed only in untreated MPS IIIB mice, the number of events was limited and no conclusions regarding an effect of resveratrol on overall survival can be drawn.

### Resveratrol Supplementation Improves Cognitive Memory and Normalizes Anxiety Levels in MPS IIIB Mice

Anxiety-related disturbances constitute a prominent feature of MPS IIIB and are also observed in the corresponding mouse model [12]. To assess these behaviors, the elevated plus maze (EPM) test was employed. The open arms represent anxiogenic, aversive conditions, whereas the closed arms provide a relatively safe and preferred environment.

**Fig. 2:**
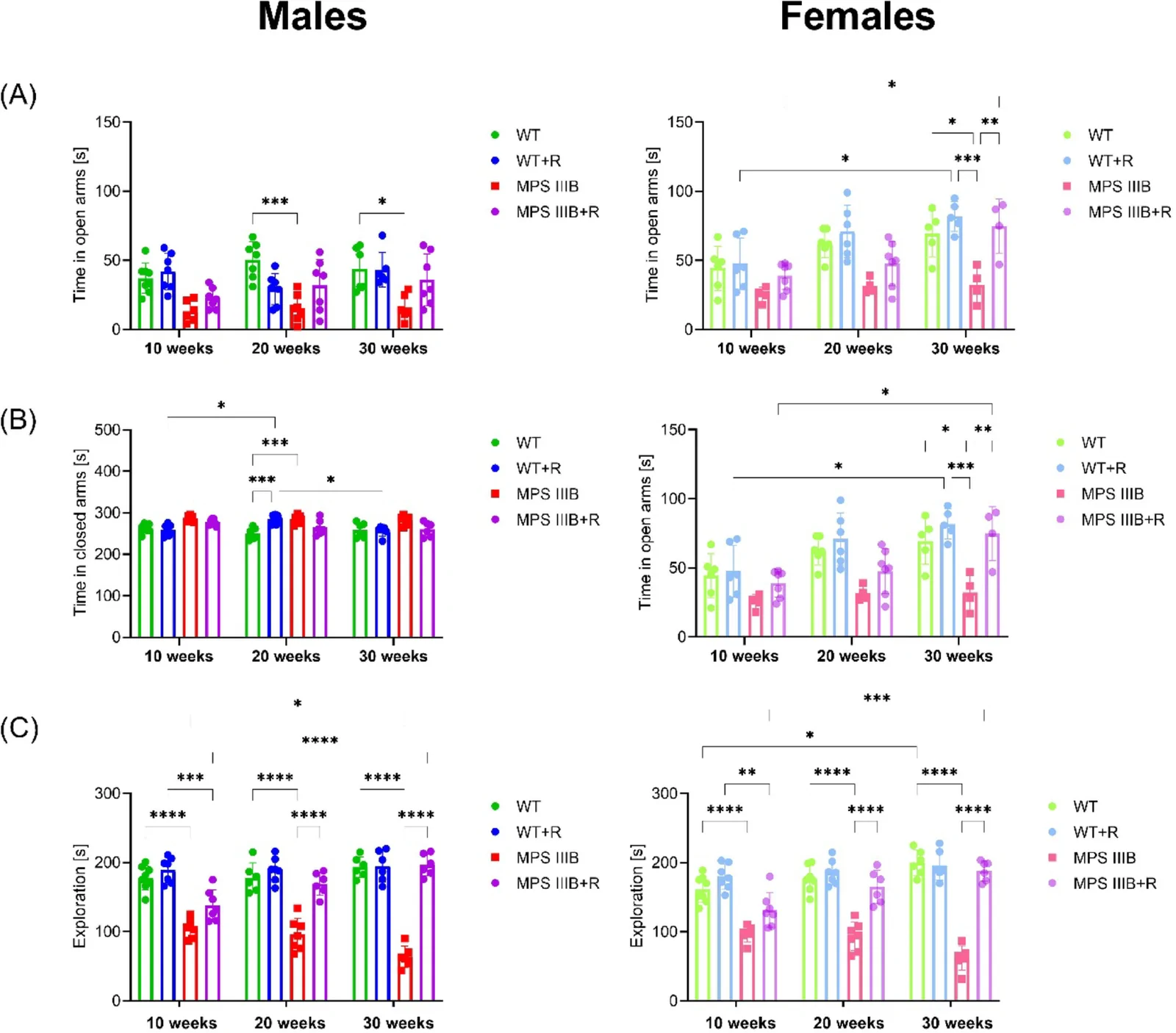
Anxiety-related behaviors in the elevated plus maze test of control (WT) and MPS IIIB mice. The following parameters were analyzed in both male and female mice: **A** time spent in open arms; **B** time spent in closed arms; and **C** exploration time. Results are shown as mean values ± SD (*n* = 5–8). Statistically significant differences are indicated by asterisks: *p < 0.05; **p < 0.01; ***p < 0.001; ****p < 0.0001

At 10 weeks of age, no differences between groups were observed in the time spent in either the open or closed arms (Fig. 2A, B). However, MPS IIIB mice showed reduced overall exploration of the elevated plus maze (EPM) compared to their wild-type (WT) littermates (Fig. 2C). From 20 weeks of age, male MPS IIIB mice spent less time in the open arms and more time in the closed arms relative to WT mice. A similar pattern was observed in female mice beginning at 20 weeks, reaching statistical significance at 30 weeks. Notably, resveratrol treatment restored this behavior to WT levels. Moreover, resveratrol-treated female mice, both WT and MPS IIIB, spent significantly more time in the open arms at 30 weeks than at 10 weeks. Additionally, overall exploration was normalized to WT levels following resveratrol treatment in both male and female mice starting from 20 weeks of age.

**Fig. 3:**
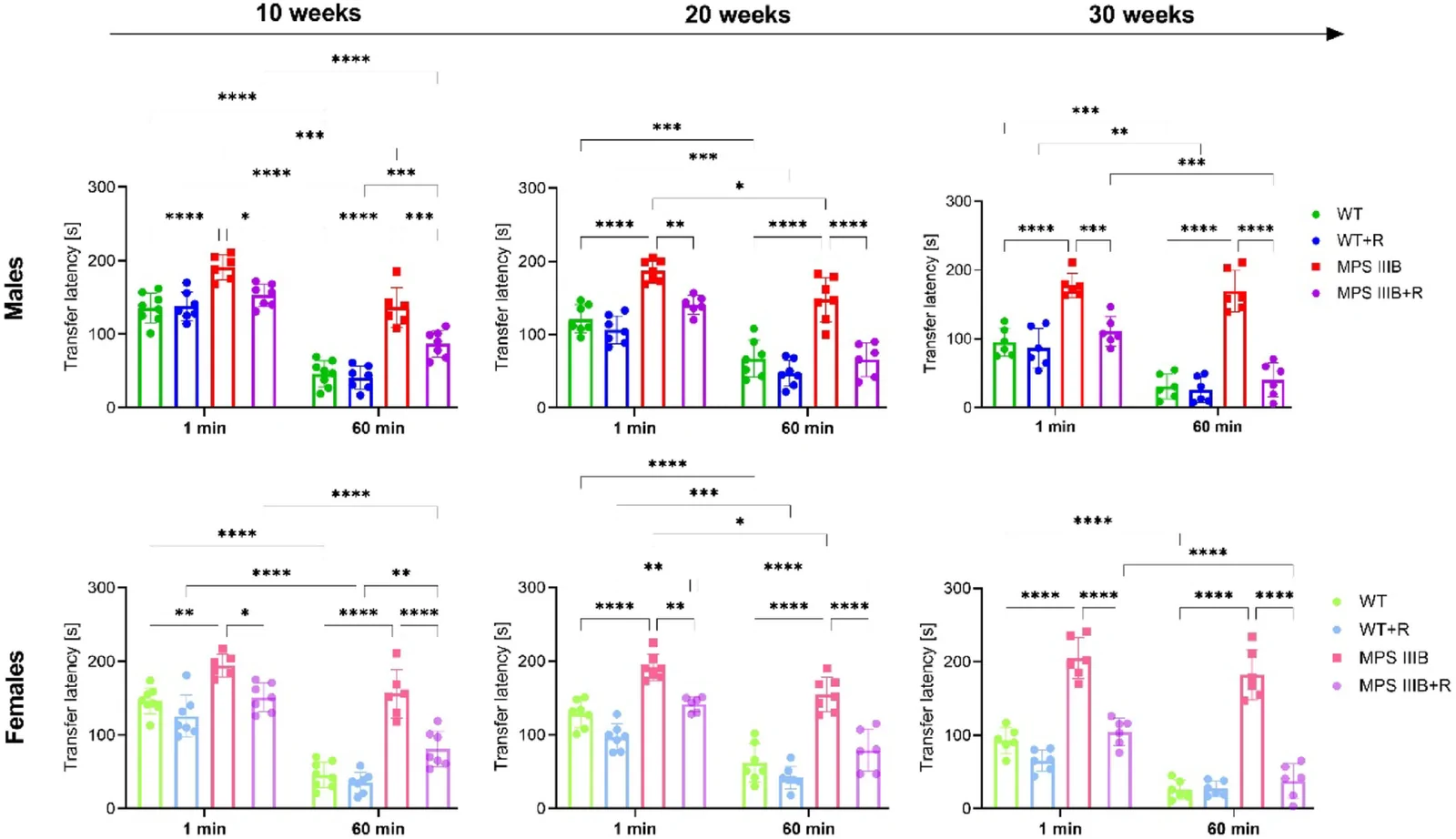
Short-term memory was assessed using the elevated plus maze test in control (WT) and MPS IIIB mice. Transfer latency was analyzed in both **A** male and **B** female mice at 1 min and again 60 min later, across three time points: 10, 20, and 30 weeks. Results are shown as mean values ± SD (*n* = 5–8). Statistically significant differences are indicated by asterisks: *p < 0.05; **p < 0.01; ***p < 0.001; ****p < 0.0001

In addition to anxiety-like behavior, the EPM can be used to evaluate short-term memory by repeating the test after a 60-minute interval. Although the EPM allows indirect assessment of memory via transfer latency, results may be influenced by anxiety levels. Transfer latency was higher in MPS IIIB mice than in WT mice in both males and females (Fig. 3). Unlike WT mice, which showed improvement over time, MPS IIIB mice did not exhibit a reduction in transfer latency across weeks, suggesting impaired short-term memory. Notably, resveratrol treatment reduced transfer latency to levels comparable to those observed in WT mice.

**Fig. 4:**
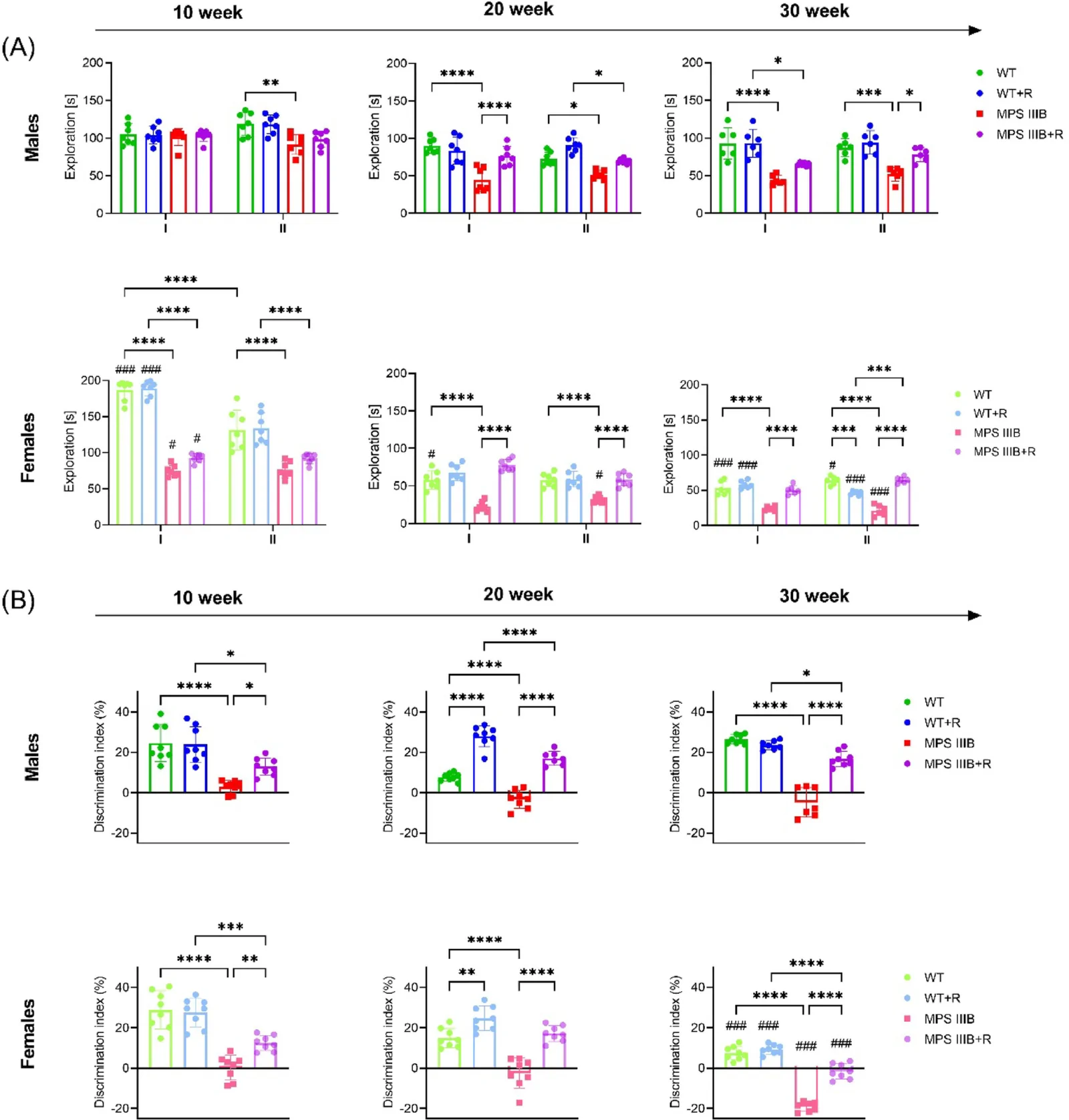
Learning and memory in MPS IIIB mice are improved following resveratrol treatment. The novel object recognition (NOR) test was used to assess **A** exploration of a novel object and **B** the discrimination index in MPS IIIB and WT mice, across three time points: 10, 20, and 30 weeks. Results are shown as mean values ± SD (*n* = 5–8). Statistically significant differences are indicated by asterisks: *p < 0.05; **p < 0.01; ***p < 0.001; ****p < 0.0001; differences between male and female mice are indicated by hashtag: ^#^*p* < 0.05; ^###^*p* < 0.001; ^####^*p* < 0.0001

Given the well-documented cognitive decline and increased anxiety in MPS IIIB, the novel object recognition (NOR) test was employed to assess the therapeutic effects of resveratrol on these mice. The NOR test is used to assess nonspatial memory in rodents in terms of time spent exploring objects. Temporary lesions of the hippocampus consistently impair object memory [14]. MPS IIIB animals showed a reduction in novel object exploration compared with WT mice (Fig. 4). Notably, at 30 weeks of age, females performed markedly worse than males, suggesting more pronounced degeneration of the cortex–hippocampal axis.

Resveratrol treatment improved these parameters in both sexes. In males, a significant increase in preference for the novel object was observed at all time points. In females, although a clear improvement relative to untreated animals was evident at 30 weeks, performance did not reach a positive discrimination level, as animals spent comparable amounts of time exploring the novel and familiar objects.

To further refine the behavioral analysis, total object interaction time was assessed. Treated mice spent more time in the central zone of the arena, indicating a reduction in anxiety-like behavior (Fig. 4). We detected sex differences between male and female MPS IIIB mice in both discrimination index and exploration of objects indicating sex-dependent differences in memory impairment. These behaviors were corrected with resveratrol treatment.

Interestingly, in 30-week-old MPS IIIB females, this measure exceeded control values, suggesting that the previously observed reduction in novel object exploration was more likely attributable to memory impairment rather than elevated anxiety.

### Resveratrol Modulates the Blood Biochemical Profile and Affects Inflammatory Response, Oxidative Transport, and Homeostasis in MPS IIIB Mice

**Fig. 5:**
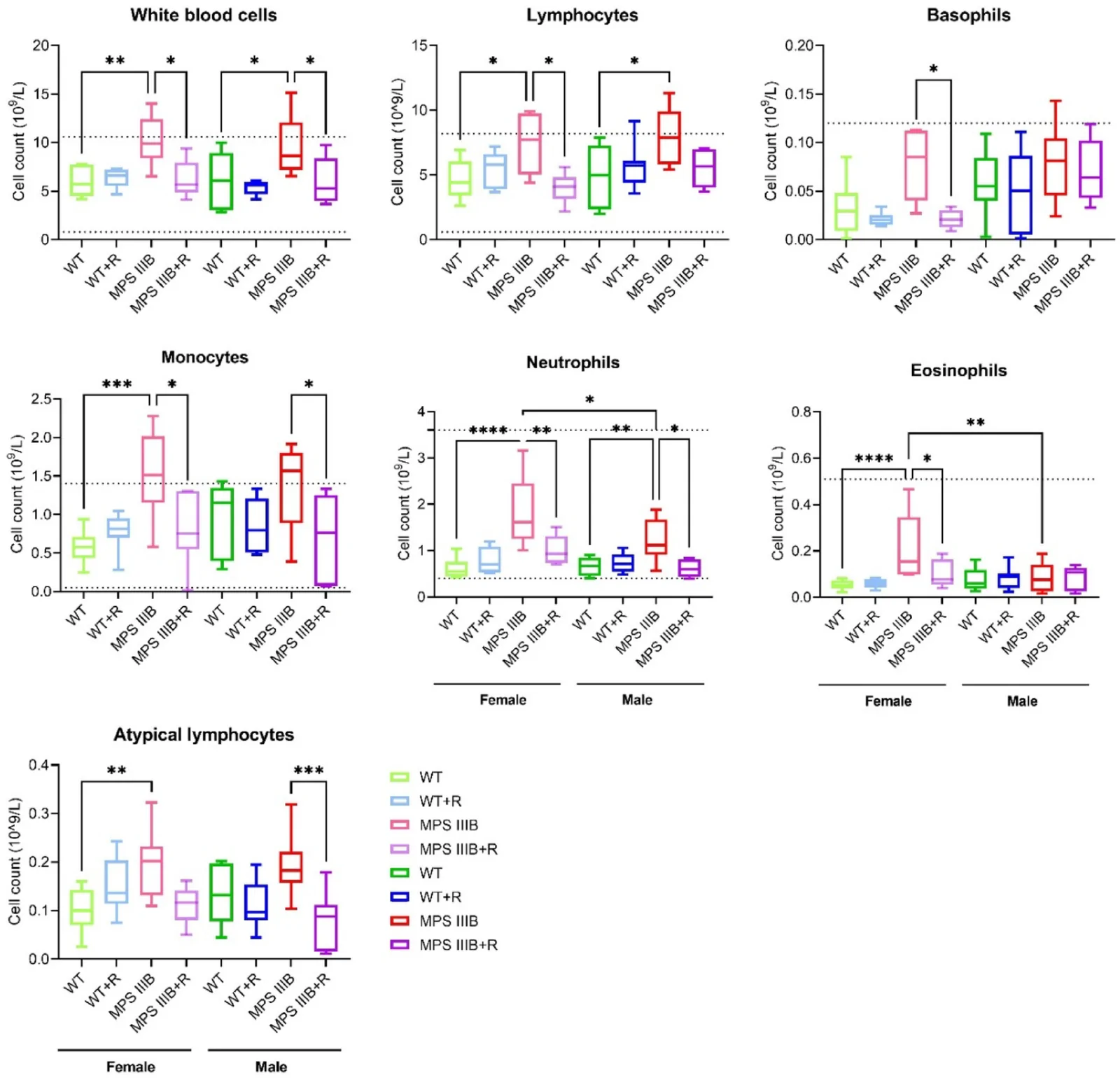
Resveratrol modulates immune-related hematological parameters in MPS IIIB mice. Hematological parameters included: white blood cells, lymphocytes, basophils, monocytes, neutrophils, eosinophils, and atypical lymphocytes. Dotted lines indicate reference values for healthy mice. Results are shown as mean values ± SD (*n* = 6–10). Statistically significant differences are indicated by asterisks: *p < 0.05; **p < 0.01; ***p < 0.001; ****p < 0.0001

Due to the generalized inflammatory state associated with MPS IIIB, hematological parameters were analyzed to evaluate the effect of resveratrol on components of the immune system, erythrocyte parameters, and homeostasis. In MPS IIIB mice, an inflammatory profile was observed involving activation of both innate and adaptive immune responses (Fig. 5). Most notably, a significantly elevated leukocyte count was recorded in both male and female MPS IIIB mice, exceeding reference values. Within the innate immune response, changes were more pronounced in female MPS IIIB mice, who showed a significant increase in neutrophils and eosinophils, as well as monocyte levels exceeding reference values. In males, a significant increase in neutrophils and elevated monocyte counts above physiological levels were observed. Within the adaptive immune response, both groups exhibited a significant increase in lymphocyte numbers beyond the normal range. The number of atypical lymphocytes was also elevated; however, statistical significance was reached only in female MPS IIIB mice in comparison to WT mice. Resveratrol treatment led to normalization of leukocyte parameters, reducing their values to levels comparable to control groups or within the physiological range in both sexes. Additionally, a decreasing trend in atypical lymphocyte counts was observed, particularly pronounced in males. These results indicate normalization of the inflammatory profile in both sexes following treatment.

Analysis of erythrocyte parameters suggested the presence of erythropoietic stress in MPS IIIB mice (Fig. S2A), as indicated by an increasing trend in nucleated erythrocyte counts, possibly associated with the inflammatory state. Moreover, males exhibited features of anemia, including significant decreases in erythrocyte count, hemoglobin concentration, and hematocrit. Resveratrol significantly reduced the number of nucleated erythrocytes in male MPS IIIB mice, whereas in female MPS IIIB mice only a decreasing trend and reduced variability were observed. In MPS IIIB males, erythrocyte and hemoglobin levels did not fully normalize to control or physiological values after treatment, although partial improvement was noted. Additionally, changes in platelet counts were observed (Fig. S2B). In MPS IIIB males, a significant increase in platelet count was detected, although still within the reference range. Following resveratrol treatment, these values significantly decreased.

Due to the multisystem nature of the disease, biochemical parameters were analyzed to determine whether the effects of resveratrol observed at the cellular level translate into changes in overall organism function. In MPS IIIB mice, a significant increase in globulin concentration was observed. This effect was particularly pronounced in females and was accompanied by a decrease in the albumin/globulin (A/G) ratio (Fig. S3A). Following resveratrol treatment, a sex-dependent effect was noted. In females, globulin levels showed a downward trend toward values observed in the control group; however, this did not result in normalization of the A/G ratio. In males, resveratrol treatment led to a significant increase in globulin concentration, accompanied by a further decrease in the A/G ratio and a marked elevation in total protein levels exceeding reference values (Fig. S3A). These findings indicate sex-dependent modulation of the immune response. Evaluation of liver markers (Fig. S3B) in females revealed a significant, though still within the reference range, increase in AST and ALP activity, with no changes in ALT. This may indicate potential early damage to hepatocytes or other tissues (skeletal muscle, heart, or brain). In affected males, no significant deviations from the control group were observed. Following resveratrol treatment, AST and ALP levels in females normalized to values close to controls, with an additional decrease in AST, suggesting a beneficial effect of the therapy on tissue homeostasis. In males, however, treatment resulted in a significant, though still within the reference range, increase in AST, along with a significant elevation in ALP and total bilirubin, which may indicate a shift toward a cholestatic profile; this requires further analysis of bilirubin fractions. Analysis of renal markers (Fig. S3C) revealed a significant increase in urea concentration above the normal range in females, and a significant decrease in creatinine concentration in males. Resveratrol treatment led to a reduction in creatinine levels in both sexes. Additionally, a significant increase in urea concentration was observed in males, while no normalization was seen in females. However, these findings do not clearly indicate impaired renal function, due to the absence of increased creatinine levels, suggesting a metabolic rather than renal origin of the observed changes. Albumin levels remained within the physiological range, excluding malnutrition as a cause of the disturbances. Additional metabolic alterations were observed (Fig. S3D). In MPS IIIB mice, a significant reduction in glucose concentration was detected, particularly pronounced in females. In this group, an increase in total cholesterol and a decrease in triglycerides below reference values were also observed. In males, metabolic changes became more evident following resveratrol treatment, which further reduced glucose levels and increased total cholesterol concentration. Moreover, the therapy significantly lowered triglyceride levels in both affected and healthy males. No clear post-treatment changes were observed in females. These results indicate that resveratrol modulates metabolism; however, the nature of these changes does not allow us to determine whether they represent a beneficial or adverse shift in the metabolic profile.

### Resveratrol Therapy Reduces Peripheral and Central Nervous System Pro-inflammatory Cytokine Levels in MPS IIIB Mice

**Fig. 6:**
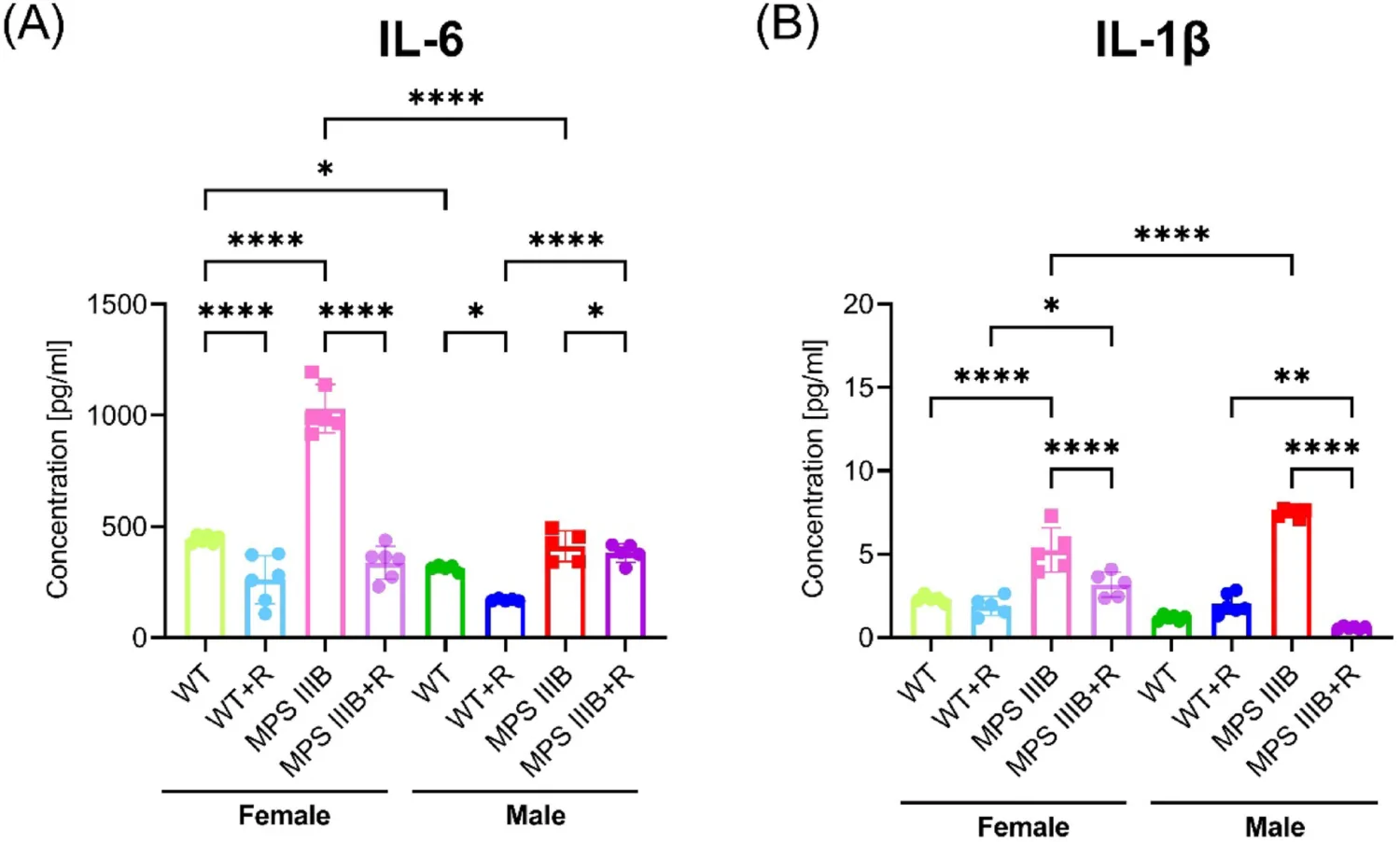
Resveratrol reduces peripheral cytokine levels in MPS IIIB mice. Levels of **A** IL-6 and **B** IL-1β. Results are shown as mean values ± SD (*n* = 6). Statistically significant differences are indicated by asterisks: *p < 0.05; **p < 0.01; ****p < 0.0001

Inflammation is one of the key pathological features of MPS IIIB. Our hematological and biochemical data described above show a clear inflammatory state in MPS IIIB mice (Figs. 5, S2, S3). We further evaluated both peripheral and central nervous system inflammation by measuring IL-6 and IL-1β concentrations. MPS IIIB mice had elevated levels of both pro-inflammatory cytokines (Fig. 6). Interestingly, females exhibited significantly higher IL-6 levels (a more humoral response) than males, in whom the increase in this cytokine did not reach statistical significance. A decrease following resveratrol treatment was observed in both groups; however, only in females did the levels return to control values, suggesting potentially more effective suppression of inflammation. Conversely, IL-1β concentrations were similarly elevated in both sexes, with a more pronounced increase in males. Additionally, resveratrol supplementation more effectively suppressed IL-1β in males than in females, the opposite of the trend seen for IL-6.

**Fig. 7:**
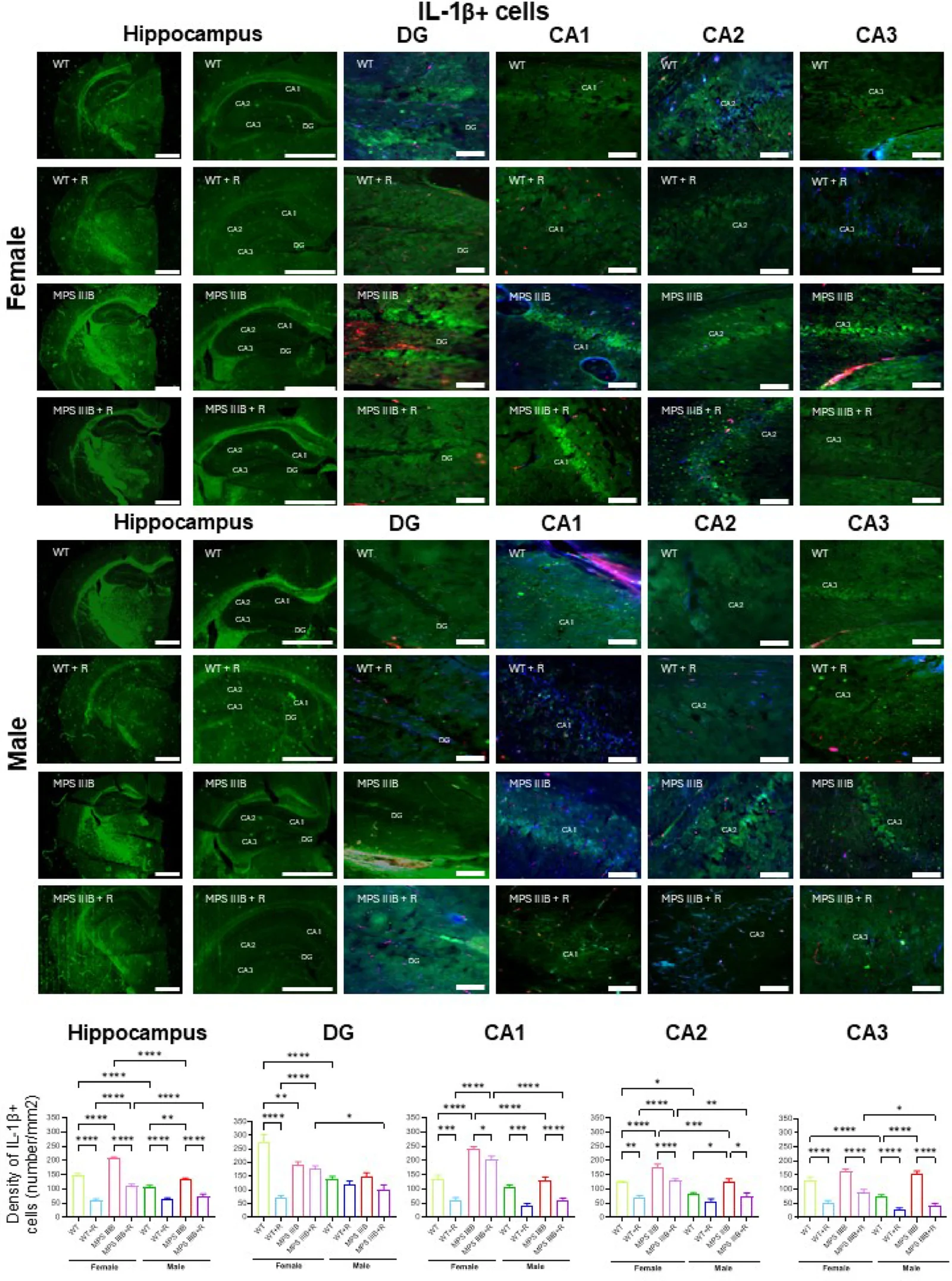
Brain immunostaining analysis of the hippocampus and its subregions showing IL-1β in female and male MPS IIIB mice. Quantification of **IL-1**β-positive cells was performed in four hippocampal subregions: CA1 (Cornu Ammonis 1), CA2 (Cornu Ammonis 2), CA3 (Cornu Ammonis 3), DG (dentate gyrus). The total number of positive cells across all analyzed regions is presented as the overall hippocampal count. Data are expressed as mean ± SEM (*n* = 7–8 mice per group). Scale bar = 100 μm. Statistically significant differences are indicated by asterisks: *p < 0.05; **p < 0.01; ***p < 0.001; ****p < 0.0001

Immunological alterations were also detected in brain structures. Behavioral analyses suggested dysfunction within the cortex–hippocampus axis and anxiety-like behavior in MPS IIIB mice [12] (Figs. 2, 3 and 4). Therefore, inflammatory markers were evaluated in hippocampal regions associated with memory and emotional regulation (Figs. 7, S4). In the hippocampus, MPS IIIB mice exhibited elevated IL-1β levels compared with WT animals, with stronger increases observed in female than in male MPS IIIB mice (Fig. 7). In contrast, peripheral inflammation was characterized mainly by elevated IL-6 in female MPS IIIB mice (Fig. 6). Resveratrol treatment significantly reduced IL-1β and IL-6 levels, although complete normalization was achieved primarily in males. The remaining elevation of cytokine levels in females indicates a less complete anti-inflammatory response to resveratrol. Analysis of hippocampal subregions revealed region- and sex-dependent differences. In CA1 and CA2, female MPS IIIB mice showed increased IL-1β expression, while IL-6 elevation was mainly detected in CA2. Resveratrol reduced inflammatory markers in both sexes, with more pronounced reductions observed in males. In CA3, increased IL-1β was observed only in MPS IIIB males and was normalized after treatment. In the dentate gyrus (DG), no major IL-1β alterations were detected, whereas IL-6 levels were elevated in male MPS IIIB mice and reduced by resveratrol supplementation. Neuronal activity, assessed by c-Fos expression, also showed regional differences. Increased c-Fos levels were detected mainly in male MPS IIIB mice, particularly in CA1 and CA3, and were reduced following resveratrol treatment. In contrast, no significant changes in neuronal activity were observed in the DG region.

**Fig. 8:**
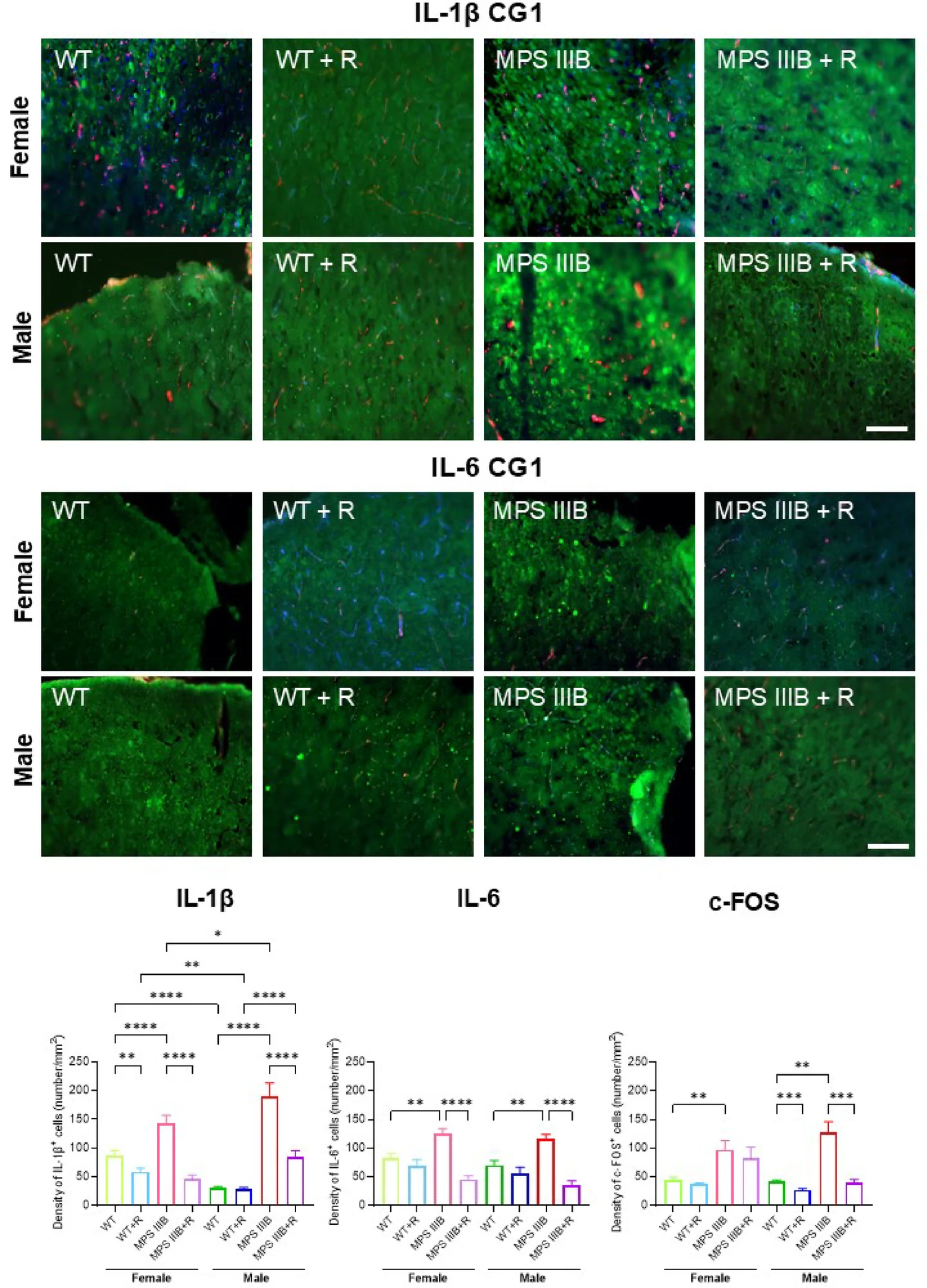
Immune response in the cingulate cortex, area 1 (CG1). The upper panel shows representative images of c-Fos-positive nuclei (red), IL-1β or IL-6 immunoreactivity (green), and DAPI staining (blue) in the CG1 region of female and male mice. The lower panel shows quantitative analysis of IL-1β-, IL-6-, and c-Fos-positive cells. Data are expressed as mean ± SEM (*n* = 7–8). Scale bar = 100 μm. Statistically significant differences are indicated by asterisks: *p < 0.05; **p < 0.01; ***p < 0.001; ****p < 0.0001

In the cerebral cortex, clear alterations in pro-inflammatory cytokine expression were observed, although the pattern differed from that detected in the hippocampus. IL-1β levels were elevated in both female and male MPS IIIB mice, with a more pronounced increase in males (Figs. 8, S5). Resveratrol treatment reduced IL-1β expression only in male MPS IIIB mice, although values did not fully normalize. No therapeutic effect was observed in females. In contrast, IL-6 levels did not differ significantly between diseased and control animals, although resveratrol supplementation reduced IL-6 expression in males. Increased neuronal activity was detected in the cortex of both sexes; however, resveratrol reduced this effect only in males (Fig. S6). Regional cortical analysis revealed distinct sex- and region-dependent inflammatory profiles. In the CG1 region, IL-1β, IL-6, and c-Fos expression were elevated in both sexes and normalized following resveratrol treatment. In CG2, inflammatory changes were more prominent in males, with elevated IL-1β and IL-6 levels reduced after therapy, while no major c-Fos alterations were observed. In the retrosplenial cortex (RSG and RSA), inflammatory responses varied between sexes. Male MPS IIIB mice showed increased IL-1β levels that decreased after resveratrol treatment, whereas females displayed more variable cytokine responses. Increased neuronal activity in these regions was particularly evident in females, especially following treatment (Figs. S5–S6). The strongest inflammatory alterations were detected in motor cortex regions (M1 and M2). IL-1β expression was elevated in both sexes, especially in males, and resveratrol reduced these levels primarily in diseased males. In females, IL-6 levels remained elevated despite treatment. Neuronal activity in M1 and M2 was increased in MPS IIIB mice and generally decreased following resveratrol supplementation, particularly in males (Figs. S5–S6).

In the amygdala, three subregions were analyzed: the basolateral amygdala (BLA), central amygdala (Ce), and bed nucleus of the stria terminalis (BST), in addition to the total number of positive cells across the entire structure. Overall IL-1β expression was elevated in both female and male MPS IIIB mice compared with WT controls and was not fully normalized by resveratrol treatment. However, regional analysis revealed clear sex-dependent differences. In the BLA, IL-1β levels were markedly higher in male MPS IIIB mice than in females, whereas in the Ce and BST regions the inflammatory response was more pronounced in females. Resveratrol treatment effectively reduced IL-1β levels in the Ce and BST of female MPS IIIB mice but showed little effect in males.

No significant differences in total IL-6 expression were observed between MPS IIIB and WT mice. However, within specific subregions, elevated IL-6 levels were detected in the BLA of female MPS IIIB mice and were normalized following resveratrol supplementation. In general, resveratrol reduced IL-6 expression across all amygdala subregions. Neuronal activity, assessed by c-Fos expression, was increased in MPS IIIB mice compared with WT controls and decreased after resveratrol treatment. In the BLA, c-Fos expression was particularly elevated in males, while resveratrol reduced neuronal activation in both sexes. In the Ce region, neuronal activity was higher in females and decreased after treatment only in female mice. In the BST, strong c-Fos elevation was observed in both sexes without significant sex differences, and resveratrol treatment effectively reduced neuronal activation in this region (Fig. S7).

## Discussion

Mucopolysaccharidosis type IIIB (MPS IIIB) is a severe pediatric neurodegenerative disorder for which no approved effective therapy currently exists. This limitation is largely due to the inability of treatments successfully used in other MPS subtypes to efficiently target the central nervous system (CNS), despite neurological dysfunction being the primary hallmark of Sanfilippo syndrome [15]. Current therapeutic strategies therefore focus on overcoming the blood–brain barrier, including enzyme modifications designed to improve CNS delivery [16]. Increasing evidence also indicates that neuroinflammation plays a major role in MPS IIIB pathogenesis and progression, contributing directly to neurodegeneration. Consequently, therapies with immunomodulatory and neuroprotective properties may represent an important complementary approach. We previously demonstrated that resveratrol reduced pro-inflammatory cytokines, increased anti-inflammatory markers, and improved behavioral outcomes in MPS IIIB mice, providing the basis for the present study [12].

The current results indicate that resveratrol improves selected behavioral outcomes and modulates neuroinflammatory markers in MPS IIIB mice. Increased mortality was observed exclusively in untreated diseased animals, whereas no deaths occurred in the resveratrol-treated groups. Previous studies reported that resveratrol extended survival in mice fed a high-calorie diet, although no significant lifespan extension was observed under standard dietary conditions, indicating that the beneficial effects of this compound may become particularly evident under physiological stress [17].

Behavioral analyses confirmed the presence of cognitive and anxiety-related disturbances in MPS IIIB mice. The novel object recognition (NOR) test may reflect deficits in hippocampus- and cortex-dependent memory functions, as healthy mice naturally prefer exploring novel stimuli [18, 19]. However, the observed behavioral alterations could also partially result from anxiety toward novel objects. This interpretation is supported by reduced exploration of the central arena during disease progression, similarly to observations reported previously in the open-field test, where MPS IIIB mice avoided the center area, suggesting anxiety-like behavior [12]. In contrast, Langford-Smith et al. (2011) did not report increased anxiety-like behavior in MPS IIIB mice but instead observed pronounced hyperactivity [20]. However, these findings may not necessarily contradict the present results, as anxiety can manifest as hyperlocomotion, potentially masking classical anxiety-like behaviors [21]. Furthermore, methodological differences may have contributed to the discrepancies between studies. In particular, the open-field arena used by Langford-Smith et al. was smaller, which may have reduced the anxiogenic properties of the test due to the limited open space available for exploration. Importantly, the absence of significant differences between the first and second exposures in the NOR task suggests that the observed behavioral phenotype cannot be attributed solely to neophobia, but likely also involves deficits in memory processing.

Resveratrol supplementation improved cognitive performance and reduced anxiety-related behaviors, suggesting favorable effects on behavioral outcomes associated with hippocampal and cortical function. Similarly, anxiolytic effects of resveratrol have been reported in mice displaying post-traumatic stress disorder-like behavior, where the authors also described potential neuroprotective effects [22, 23]. Nevertheless, memory deficits were not fully reversed. Importantly, therapeutic responses differed between sexes, which may result not only from physiological differences but also from distinct baseline severity of disease manifestations. Even significant improvement may not lead to complete normalization in animals presenting more severe initial pathology.

Sex-dependent variability may additionally reflect differences in behavioral phenotype and disease progression. Female MPS IIIB mice display more submissive behavior, whereas males more frequently exhibit aggression, suggesting differential involvement of specific brain regions [24]. Consequently, resveratrol may affect distinct dominant pathological mechanisms in males and females.

The behavioral alterations observed in MPS IIIB mice may be linked to inflammatory activation [12], which is supported by hematological findings. Diseased mice exhibited leukocytosis, indicating systemic inflammation involving changes in both innate and adaptive immune cell populations, including neutrophils and lymphocytes. Interestingly, females displayed more pronounced changes in several innate immune cell populations, whereas elevated platelet counts in males may reflect compensatory regulation and tissue repair processes, as platelets are also involved in vascular and tissue regeneration [25]. Signs of erythropoietic stress and anemia were particularly pronounced in males in our study. Reduced hemoglobin and erythrocyte counts accompanied by mildly elevated platelet levels in MPS IIIB rodents were also observed in other studies [26].

Resveratrol effectively reduced multiple inflammatory hematological parameters, further supporting its anti-inflammatory activity. However, atypical lymphocyte levels remained elevated in females, suggesting incomplete normalization of the inflammatory profile. Moreover, changes in total lymphocyte populations do not clearly indicate whether immune responses shift toward pro-inflammatory or regulatory phenotypes. Previous studies demonstrated that resveratrol may modulate lymphocyte balance by reducing pro-inflammatory Th17 responses and promoting regulatory Treg populations [27]. In males, however, the reduction of platelet counts below physiological levels after treatment raises the question of whether excessive suppression could impair regenerative processes. Interestingly, a case of thrombocytopenia associated with resveratrol supplementation has been reported [28].

Biochemical analyses further confirmed systemic dysregulation in MPS IIIB mice. Elevated globulin concentrations supported the presence of generalized inflammation. Increased AST and ALP activity in females may indicate tissue damage, although the absence of ALT elevation suggests that tissues other than the liver, including skeletal muscle, heart, or brain, may contribute to these alterations [29]. Increased urea levels without changes in creatinine likely reflect metabolic dysregulation rather than direct renal injury, consistent with reports describing urea cycle disturbances in MPS IIIB mice [30]. Disturbances in glucose and lipid metabolism were also observed, particularly in females, suggesting impaired energy homeostasis. Similar metabolic suppression involving carbohydrate and lipid depletion was previously described in MPS IIIB mice [31].

Interestingly, resveratrol produced opposite effects on globulin levels depending on sex, decreasing them in females while increasing them in males. Since globulins include both pro-inflammatory and regulatory immune components, interpretation remains limited without detailed fraction analysis. Nevertheless, when considered together with hematological and behavioral findings, these changes may reflect beneficial immune modulation. Resveratrol also influenced metabolic parameters, reducing glucose levels and triglycerides, particularly in males, potentially reflecting a metabolic profile similar to caloric restriction. Similar metabolic effects, including improved glucose and lipid homeostasis accompanied by extended survival, have previously been reported in mice treated with resveratrol [32].

Both ELISA and immunofluorescence analyses confirmed widespread inflammatory activation in the CNS and peripheral tissues of MPS IIIB mice. The inflammatory profile differed between sexes, with a stronger IL-6 response observed in females, whereas males showed a less pronounced increase in this cytokine. These sex differences may result from distinct hormonal regulation [33]. Previous studies have shown that females exhibit a stronger IL-6-dependent inflammatory response to stressors, while testosterone reduces TLR4 receptor expression on macrophages. This may partly explain the lack of IL-6 elevation in males, as its production is strongly associated with TLR4-dependent signaling pathways. In contrast, IL-1β can also be produced through inflammasome activation [34]. Distinct sex-dependent neuroinflammatory patterns were also identified within the hippocampus and cortex. In females, inflammatory responses were especially pronounced in CA1 and cortical regions involved in emotional memory, spatial orientation, and anxiety processing, including RSA and RSG. Inflammatory alterations were also observed in motor regions (M1/M2), suggesting possible effects on motor function. In males, inflammation predominantly affected CA3 and the dentate gyrus, regions associated with memory integration, pattern separation, and neurogenesis. Such alterations may impair information processing and stress adaptation. Males additionally displayed inflammatory changes in CG2, a region associated with cognitive flexibility and decision-making. In females, inflammatory alterations were particularly evident in CA2, a region linked to social memory and aggression regulation, whereas in males they were more pronounced in CG1, associated with cognitive control and behavioral adaptation.

Resveratrol therapy effectively alleviated peripheral inflammation by reducing the levels of all elevated pro-inflammatory markers. These findings are consistent with the previous study [12], in which treatment with the same polyphenol reduced TNF-α levels in both females and males. Resveratrol also exerted significant anti-inflammatory effects within both hippocampal and cortical regions, although the response was more extensive in males. In females, therapeutic effects were particularly evident in CA2 and CA3, suggesting improved social and contextual memory processing. However, some regions, such as M1 and RSG, showed persistent or even elevated inflammatory markers, indicating that resveratrol may also modulate neuronal activation and network excitability. In male MPS IIIB mice, treatment reduced inflammatory markers across most hippocampal and cortical regions and normalized excessive neuronal activity, suggesting broader effects on neuroinflammatory and neuronal responses.

Importantly, inflammation alone does not necessarily indicate ongoing structural neurodegeneration, as neuronal activation may also contribute to altered behavioral outcomes. Furthermore, behavioral studies in mice are inherently characterized by high variability due to environmental sensitivity, handling procedures, microbiota composition, and sex-related differences [35, 36]. Previous studies using the same MPS IIIB model reported inconsistent behavioral phenotypes ranging from anxiety-related hyperactivity to reduced locomotion and decreased anxiety responses [12, 37]. Recent findings suggest that these behavioral abnormalities may emerge earlier than previously thought [12], although initially in a subtle form. Sex-dependent differences were also observed, with females showing more pronounced behavioral alterations than males [37]. The present findings suggest that reduced anxiety-like behavior may reflect impaired processing of threatening stimuli rather than a true reduction in anxiety. A limitation of this study is that the cellular source of IL-1β and IL-6 was not determined. Future studies using cell-type-specific markers, such as Iba1, GFAP, and NeuN, will help clarify the cellular mechanisms underlying neuroinflammation in MPS IIIB and the effects of resveratrol.

Overall, the present study indicates that resveratrol modulates neuroinflammatory responses and improves selected behavioral outcomes in MPS IIIB mice. Despite several limitations and complex sex-dependent responses, the findings provide new insight into the inflammatory and neurodegenerative mechanisms underlying MPS IIIB and support further investigation of resveratrol as a potential therapeutic strategy targeting CNS pathology in Sanfilippo syndrome.

## Supplementary Information

Below is the link to the electronic supplementary material.

Supplementary Material 1

## Data Availability

No datasets were generated or analysed during the current study.
