# Supplementary material for: Resveratrol Improves Memory and Reduces Neuroinflammation in an MPS IIIB Mouse Model
Source: Neurochem Res. 2026 Oct 13;51(5):290. doi: 10.1007/s11064-026-04920-6 (PMC13661645; doi:10.1007/s11064-026-04920-6)
Supplement: Supplementary file 1 [file 11064_2026_4920_MOESM1_ESM.pdf]

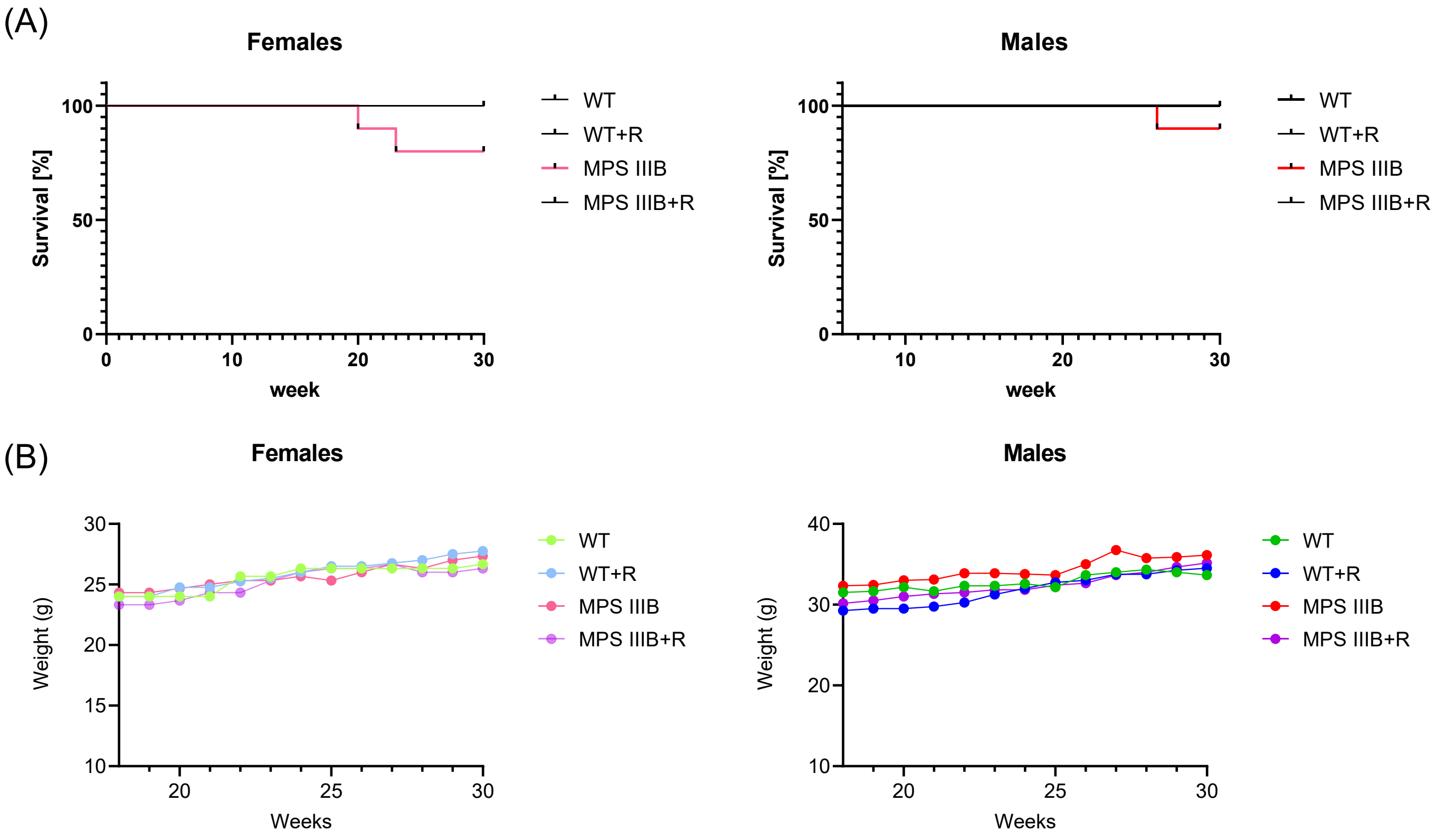

Figure S1. Resveratrol Affects Survival and Body Weight in MPS IIIB Mice (A) Survival curves (B) Body weight (N=6-10).

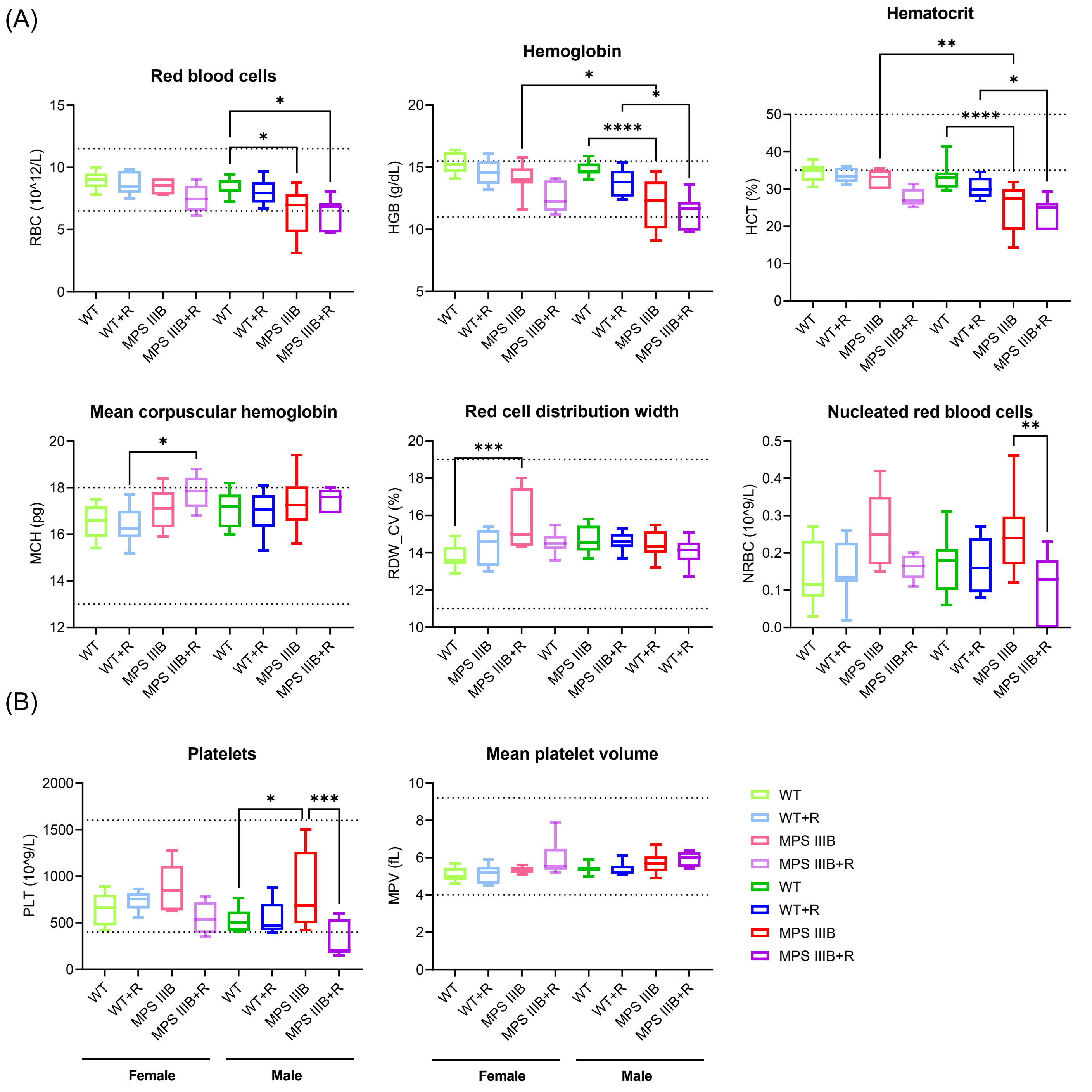

Figure S2. Oxygen transport and homeostasis in MPS IIIB. (A) Oxygen transport parameters included: Red Blood Cells, Hemoglobin, Hematocrit, Mean corpuscular hemoglobin, Red Cell Distribution width, Nucleated Red Blood Cells; and (B) Homeostasis parameters included: Plelets and Mean Platelet Volume. Dotted lines indicate references values for healthy mice. Results are shown as mean values ± SD (n=6-10). Statistically significant differences are indicated by asterisks: *$p < 0.05$; ***$p < 0.001$; ****$p < 0.0001$.

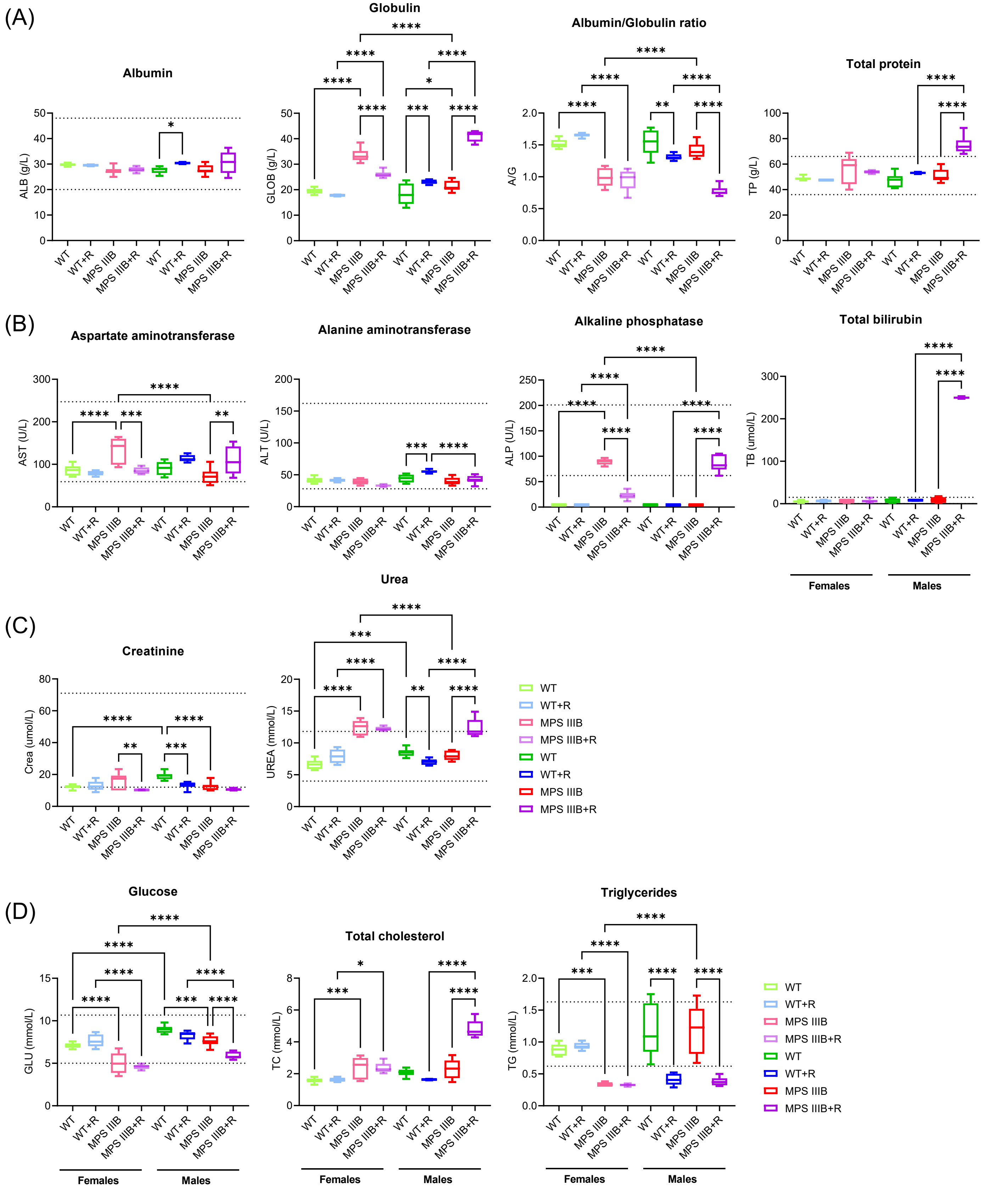

Figure S3. Biochemical analysis in MPS IIIB. (A) Serum protein profile; (B) Liver markers; (C) Renal markers; (D) Metabolic analusis. Dotted lines indicate references values for healthy mice. Results are shown as mean values ± SD (n=6-10). Statistically significant differences are indicated by asterisks: *$p < 0.05$; **$p < 0.01$; ***$p < 0.001$; ****$p < 0.0001$.

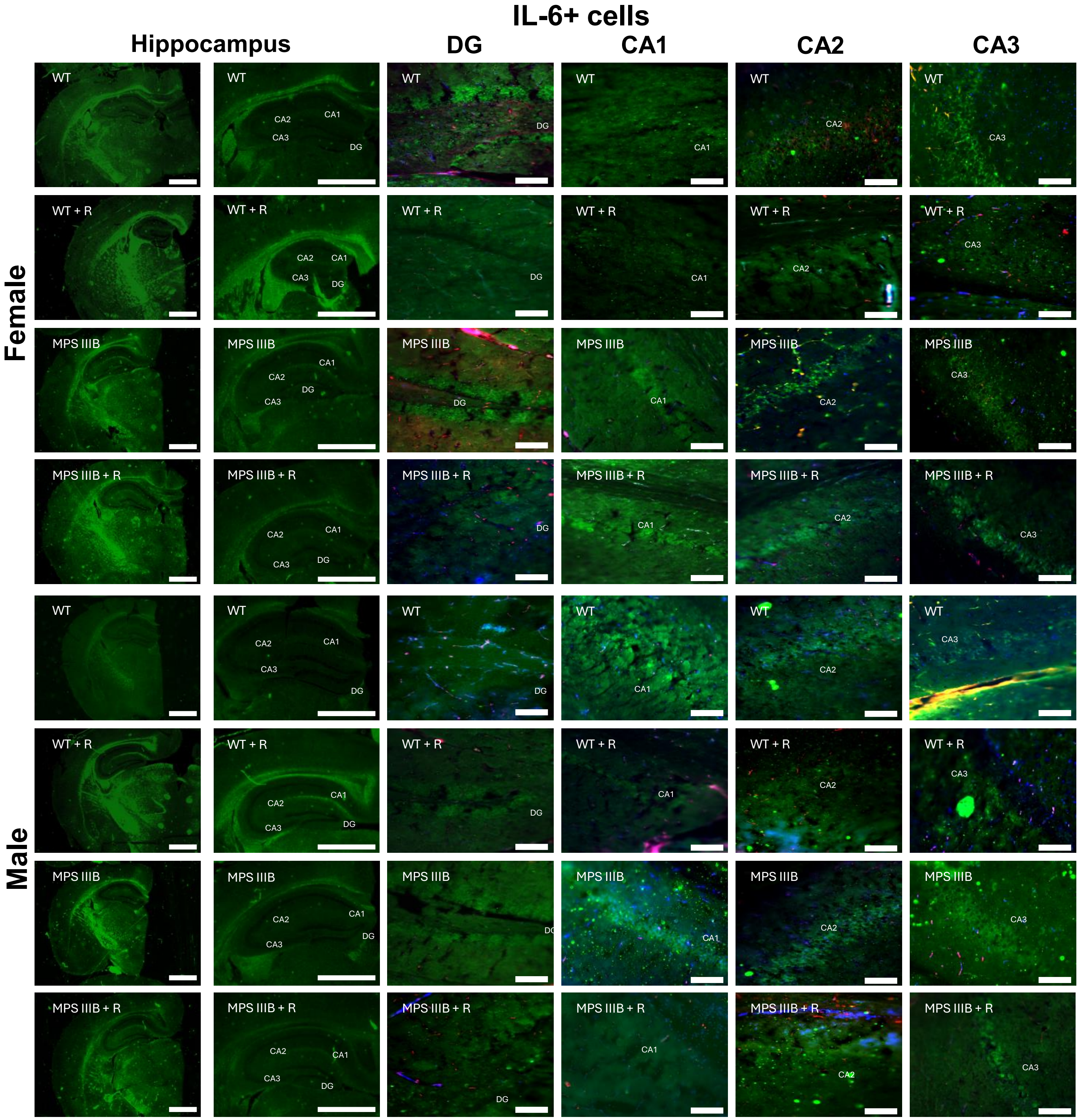

Figue S4. Brain immunostaining analysis of the IL-6-, and FOS-positive cells in hippocampus and its subregions showing immune responses in female and male MPS IIIB mice. Data are expressed as mean ± SEM (n = 7–8 mice per group). Statistically significant differences are indicated by asterisks: $*p < 0.05$; $**p < 0.01$; $***p < 0.001$; $****p < 0.0001$.

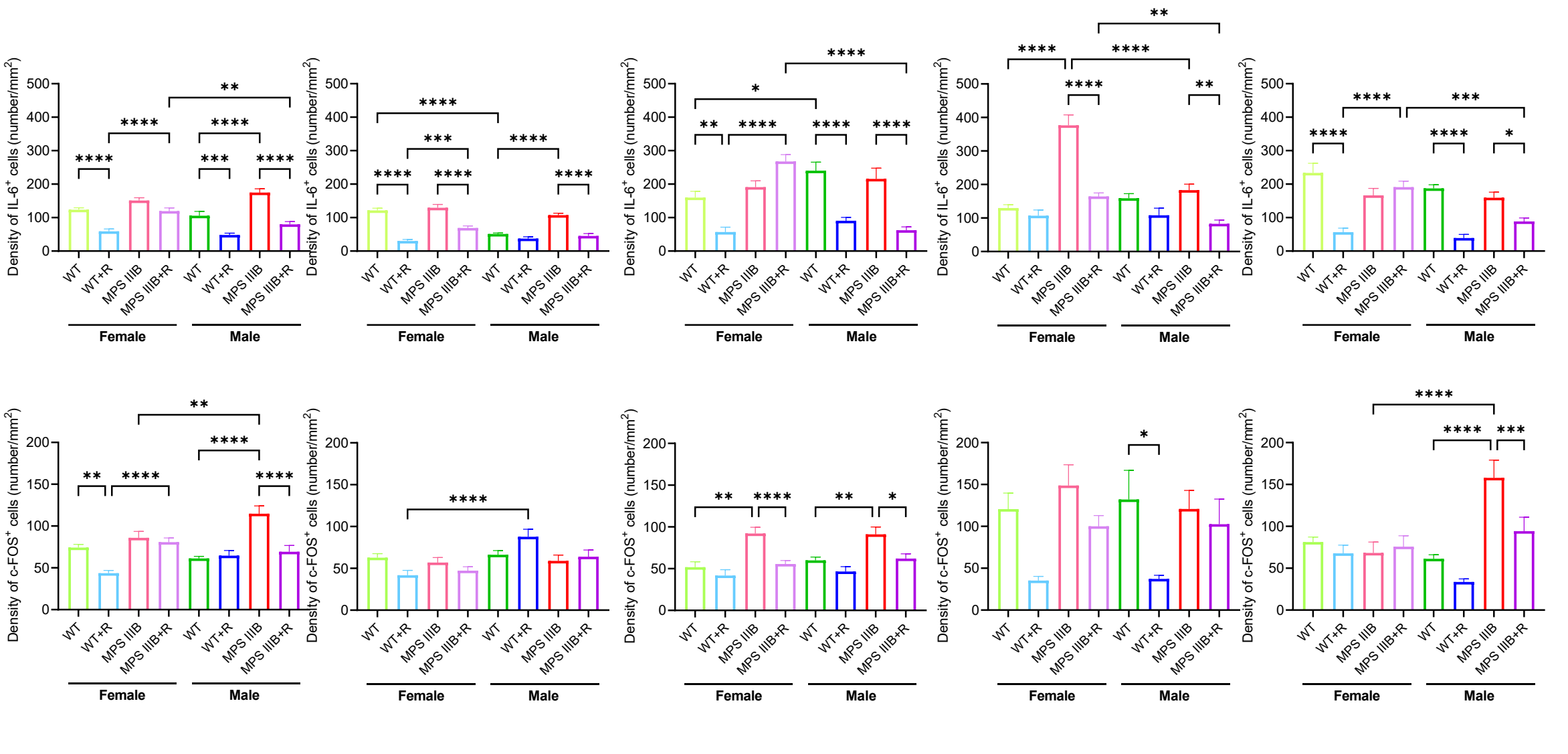

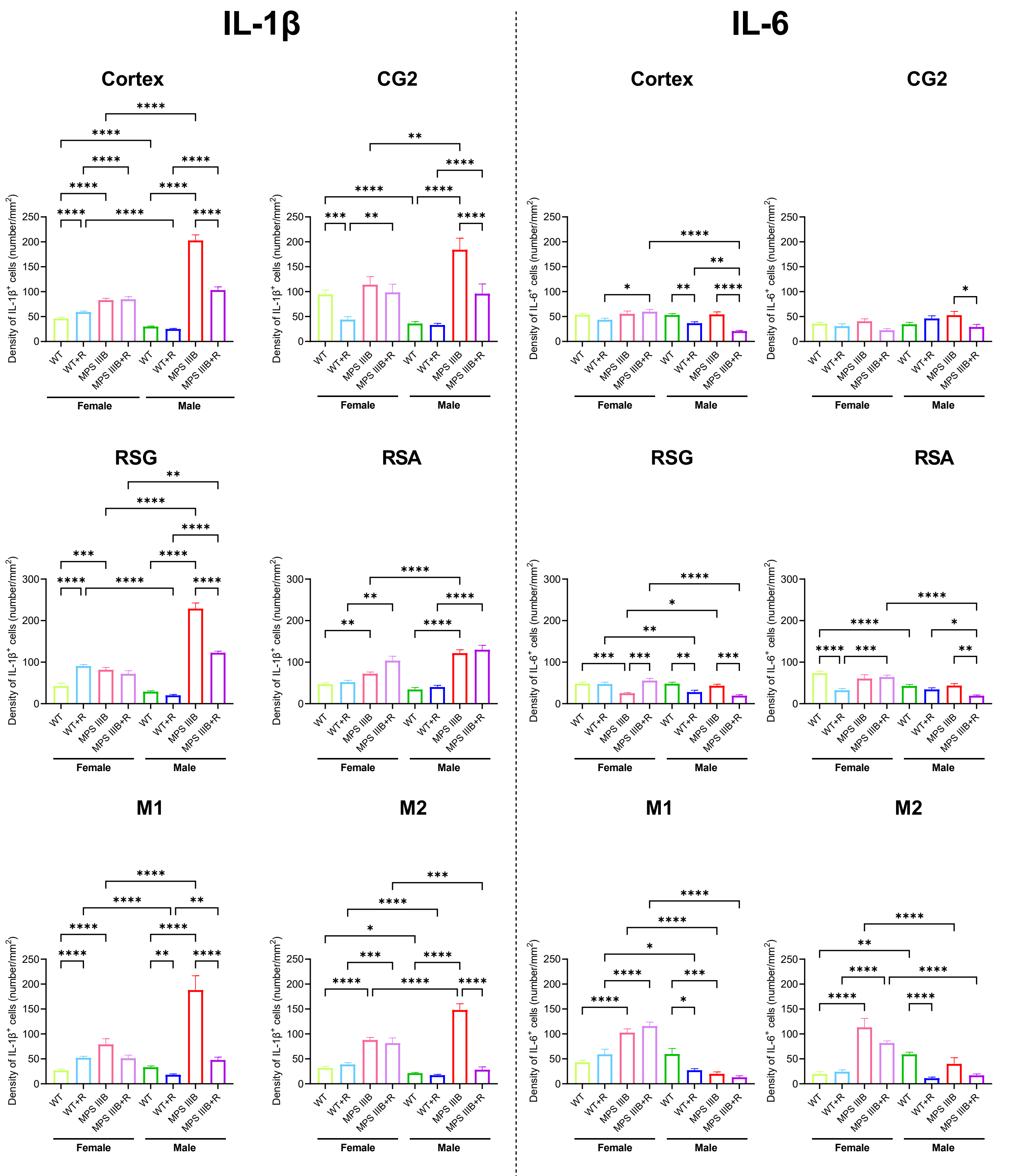

Figue S5. Brain immunostaining analysis of the cortex and its subregions showing immune responses in female and male MPS IIIB mice. Quantification of IL-1β-, IL-6-positive cells was performed in five cortex subregions: cingulate cortex, area 2 (CG2), retrosplenial granular cortex (RSG), retrosplenial agranular cortex (RSA), primary motor cortex (M1), and secondary motor cortex (M2). The total number of positive cells across all analyzed regions is presented as the overall cortex count. Data are expressed as mean ± SEM (n = 7–8 mice per group). Statistically significant differences are indicated by asterisks: **p* < 0.05; ***p* < 0.01; ****p* < 0.001; ****p < 0.0001.

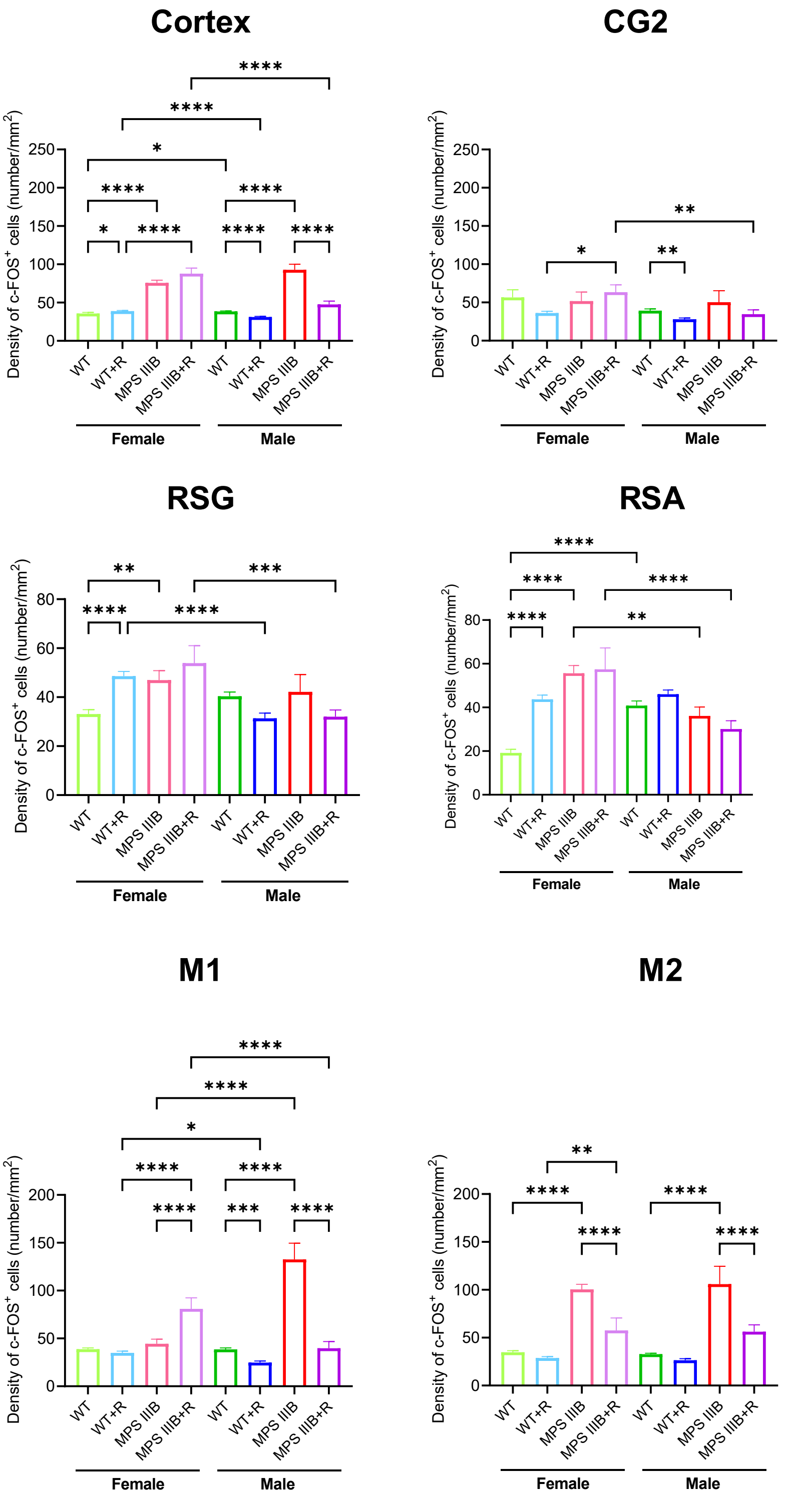

Figue S6. Brain immunostaining analysis of the cortex and its subregions showing c-FOS in female and male MPS IIIB mice. Quantification of c-FOS-positive cells was performed in five cortex subregions: cingulate cortex, area 2 (CG2), retrosplenial granular cortex (RSG), retrosplenial agranular cortex (RSA), primary motor cortex (M1), and secondary motor cortex (M2). The total number of positive cells across all analyzed regions is presented as the overall cortex count. Data are expressed as mean ± SEM (n = 7–8 mice per group). Statistically significant differences are indicated by asterisks: *$p < 0.05$; **$p < 0.01$; ***$p < 0.001$; ****p < 0.0001.

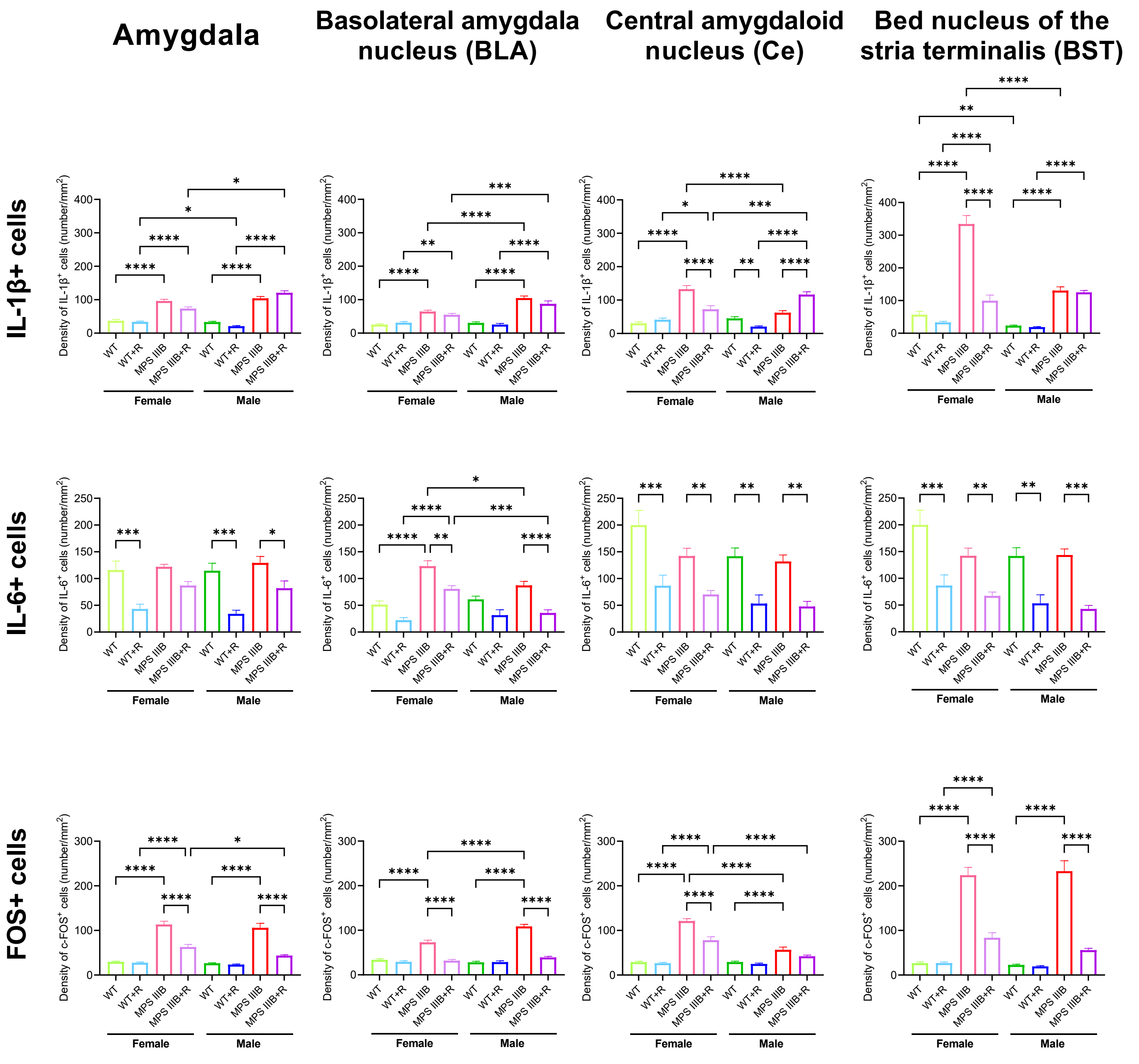

Figue S7. Brain immunostaining analysis of the amygdala and its subregions showing immune responses in female and male MPS IIIB mice. Quantification of IL-1β-, IL-6-, and FOS-positive cells was performed in three amygdala subregions: the basolateral amygdala (BLA), central amygdala (Ce), and bed nucleus of the stria terminalis (BST). The total number of positive cells across all analyzed regions is presented as the overall amygdala count. Data are expressed as mean ± SEM (n = 7–8 mice per group). Statistically significant differences are indicated by asterisks: **p* < 0.05; ***p* < 0.01; ****p* < 0.001; ****p < 0.0001.
